# Estimating Adherence Based on Prescription or Dispensation Information: Impact on Thresholds and Outcomes. A Real-World Study With Atrial Fibrillation Patients Treated With Oral Anticoagulants in Spain

**DOI:** 10.3389/fphar.2018.01353

**Published:** 2018-12-03

**Authors:** Isabel Hurtado-Navarro, Aníbal García-Sempere, Clara Rodríguez-Bernal, Yared Santa-Ana-Tellez, Salvador Peiró, Gabriel Sanfélix-Gimeno

**Affiliations:** ^1^Fundación para el Fomento de la Investigación Sanitaria y Biomédica de la Comunidad Valenciana, Valencia, Spain; ^2^Red de Investigación en Servicios de Salud en Enfermedades Crónicas, Valencia, Spain

**Keywords:** medication adherence, proportion of days covered, threshold, oral anticoagulants, clinical outcomes

## Abstract

**Objective:** To estimate drug exposure, Proportion of Days Covered (PDC) and percentage of patients with PDC ≥ 80% from a cohort of atrial fibrillation patients initiating oral anticoagulant (OAC) treatment. We employed three different approaches to estimate PDC, using either data from prescription and dispensing (PD cohort) or two common designs based on dispensing information only, requiring at least one (D1) or at least two (D2) refills for inclusion in the cohorts. Finally, we assessed the impact of adherence on health outcomes according to each method.

**Methods:** Population-based retrospective cohort of all patients with Non Valvular Atrial Fibrillation (NVAF), who were newly prescribed acenocoumarol, apixaban, dabigatran or rivaroxaban from November 2011 to December 2015 in the region of Valencia (Spain). Patients were followed for 12 months to assess adherence using three different approaches (PD, D1 and D2 cohorts). To analyze the relationship between adherence (PDC ≥ 80) defined according to each method of calculation and health outcomes (death for any cause, stroke or bleeding) Cox regression models were used. For the identification of clinical events patients were followed from the end of the adherence assessment period to the end of the available follow-up period.

**Results:** PD cohort included all patients with an OAC prescription (*n* = 38,802), D1 cohort excluded fully non-adherent patients (*n* = 265) and D2 cohort also excluded patients without two refills separated by 180 days (*n* = 2,614). PDC ≥ 80% ranged from 94% in the PD cohort to 75% in the D1 cohort. Drug exposure among adherent (PDC ≥ 80%) and non-adherent (PDC < 80%) patients was different between cohorts. In adjusted analysis, high adherence was associated with a reduced risk of death [Hazard Ratio (HR): from 0.82 to 0.86] and (except in the PD cohort) the risk for ischemic stroke (HR: from 0.61 to 0.64) without increasing the risk of bleeding.

**Conclusion:** Common approaches to assess adherence using measures based on days’ supply exclude groups of non-adherent patients and, also, misattribute periods of doctors’ discontinuation to patient non-adherence, misestimating adherence overall. Physician-initiated discontinuation is a major contributor to reduced OAC exposure. When using the PDC80 threshold, very different groups of patients may be classified as adherent or non-adherent depending on the method used for the calculation of days’ supply measures. High adherence and high exposure to OAC treatment in NVAF patients is associated with better health outcomes.

## Introduction

While it seems obvious that patients need to take their medications to benefit from them as shown in clinical trials, several studies and systematic reviews have analyzed the association between adherence to evidence-based pharmacotherapy and outcomes in chronic diseases such as coronary ischemic disease (McDermott et al., 1997; Rasmussen et al., 2007; Bitton et al., 2013; Choudhry et al., 2014), hyperlipidemia (De Vera et al., 2014; Karlsson et al., 2018), diabetes (Hood et al., 2009; Khunti et al., 2017), hypertension (Dragomir et al., 2010; Xu et al., 2017), osteoporosis (Nikitovic et al., 2010) and other conditions (DiMatteo et al., 2002; Simpson et al., 2006). Not surprisingly, virtually all the published evidence has shown that high adherence to appropriate treatment reduces mortality, several adverse clinical events, readmissions, healthcare utilization and costs. Oral anticoagulant (OAC) treatment in atrial fibrillation (AF) patients is not an exception and several studies (Deitelzweig et al., 2013; Shore et al., 2014; Thorne et al., 2014; Spivey et al., 2015; Alberts et al., 2016; Yao et al., 2016; Borne et al., 2017; Deshpande et al., 2018) have shown the valuable impact of high levels of adherence to OAC medication on various clinical outcomes, particularly the prevention of cardioembolic stroke.

Although there are several methods for assessing adherence (metabolite or biologic marker detection, self-reporting, pill count, electronic monitoring devices and other), retrospective studies based on data from refill databases have been favored in recent years because health organizations have extensively introduced health information systems with a unique patient identifier and, also, because this methodology has some advantages regarding the alternatives: it is unobtrusive (thus patient sensitization is avoided), objective (it produces quantifiable and reproducible data for each individual), and pragmatic (it is easy to use and analyze, inexpensive and applicable to different settings and drugs) (Rudd, 1979; Steiner and Prochazka, 1997; Schneeweiss and Avorn, 2005; Suissa and Garbe, 2007; Wettermark et al., 2013).

Despite the presence of an important methodological heterogeneity, studies linking adherence and health outcomes using refill databases usually adopt a common analytical approach: patients are categorized as adherent or non-adherent according to a threshold on medication days’ supply over a definite period, and the relationship between these two groups (patients under and over the threshold) and outcomes is evaluated. Generally, in the literature on adherence to OAC (Shore et al., 2014; Alberts et al., 2016; Yao et al., 2016; Borne et al., 2017; Deshpande et al., 2018) and to other chronic treatments, patients are defined as adherent when they exceed a cut-off point of 80% in the Proportion of Days Covered (PDC) or in other analogous adherence measures based on days’ supply, as the Medication Possession Ratio (MPR).

The 80% threshold (PDC80) originated in blood pressure studies carried out in the 70s of the last century (Sackett et al., 1975), but does not have an empirical basis for dichotomizing adherence to OAC treatment in AF patients, nor in many other conditions (Watanabe et al., 2013; Lo-Ciganic et al., 2015; Gellad et al., 2017; Morrison et al., 2017; Stauffer et al., 2017) [although some studies give some support to the 80% threshold in some conditions (Hansen et al., 2009; Karve et al., 2009)], introducing an element of arbitrariness with regard to the consideration of “sufficient” adherence and also to the interpretation of the relationship between adherence and outcomes.

Moreover, adherence measures based on days’ supply such as PDC or MPR can be calculated in several ways (Steiner and Prochazka, 1997; Andrade et al., 2006; Nikitovic et al., 2010; Vrijens et al., 2012; Arnet et al., 2016; Krueger et al., 2018). This in turn generates a huge variability in the real exposure to treatment of patients categorized as “adherent” or “non-adherent” using an apparently equal cut-off point value and, therefore, in the meaning of a given threshold or in the relationships between adherence (defined as PDC ≥ 80% independently of the method used for its calculation) and outcomes.

Differences in the design of adherence studies and in the operational definitions employed in these studies can importantly skew the adherence estimates based on days’ supply. In this way, the relationship between adherence thresholds and outcomes may be more sensitive to the way in which PDC (or MPR) has been calculated than to the actual patient exposure to the medication. These differences commonly arise from: (1) the exclusion (total or partial) from adherence studies of non-adherent patients (e.g., designs where two or more prescriptions filled or a minimum of covered days are required as an inclusion criteria) (Crivera et al., 2015), (2) the inclusion of patients more likely to be adherent, e.g., studies including experienced users (in the field of OAC, those can include long term users of an index drug, or switchers to a different OAC drug) (Ray, 2003; Danaei et al., 2012; Maciejewski et al., 2013; Lund et al., 2015; Li et al., 2018), (3) the definition of the index date (the date of first prescription, filled or not, or alternatively the date of the first prescription filled), (4) the censoring of cases at the date of the last refill, avoiding the use of a fixed time-window (e.g., 12 months) for follow-up and calculation of days’ supply based estimates (Kozma et al., 2013), (5) the censoring of cases (or the classification as non-adherent) when patients switch to a different treatment of the same class (Zhu et al., 2017), (6) the consideration (or not) of stockpiling (Greevy et al., 2011), (7) the consideration (or not) of immeasurable time (Dong et al., 2017; Palmaro et al., 2017), and (8) some other variants such as the estimation of the daily dose, the duration of the look-back period (Riis et al., 2015; Roberts et al., 2015; Nakasian et al., 2017), or the censoring (or not) of deceased patients or those who lose coverage.

Finally, in most adherence studies the information about doctors’ prescription is absent from refill databases. This is especially relevant because the concept of adherence refers to the “*extent to which patients take medications as prescribed by their health care providers*” (Sabaté, 2003; Osterberg and Blaschke, 2005; Cramer et al., 2008). In absence of a doctors’ prescription information, studies based on refill databases do not capture patients who do not fill any prescription (therefore, they may overestimate adherence as fully non-adherent patients are not accounted for) or may misclassify some patients as non-adherent when in fact the gap in days’ supply is caused by treatment discontinuation decided by their physicians (in this case, these studies may underestimate adherence due to the erroneous classification as non-adherent of patients not on treatment anymore).

The aims of this study, using a retrospective cohort of all patients with Non Valvular Atrial Fibrillation (NVAF) newly prescribed an OAC drug in the Valencia Region during nearly 5 years, are: (1) to estimate the variability in days’ supply adherence measures (PDC and PDC80) using a doctors’ prescription-based design and two common dispensation-based designs regarding real drug exposure, and (2) to determine the impact of adherence (defined as PDC ≥ 80%) on health outcomes according to each method of calculation of PDC.

## Materials and Methods

### Study Design

Population-based retrospective cohort of all patients aged 40 and over with NVAF, who were newly prescribed acenocoumarol, apixaban, dabigatran or rivaroxaban from November 2011 to December 2015 in the region of Valencia (Spain). Patients were followed for 12 months to assess adherence using three different approaches, and from the end of the adherence assessment period to the end of the available follow-up period to analyze the relationship between adherence (PDC ≥ 80, defined according to each method of calculation) and health outcomes (death, stroke or bleeding).

### Population and Setting

**FIGURE 1:**
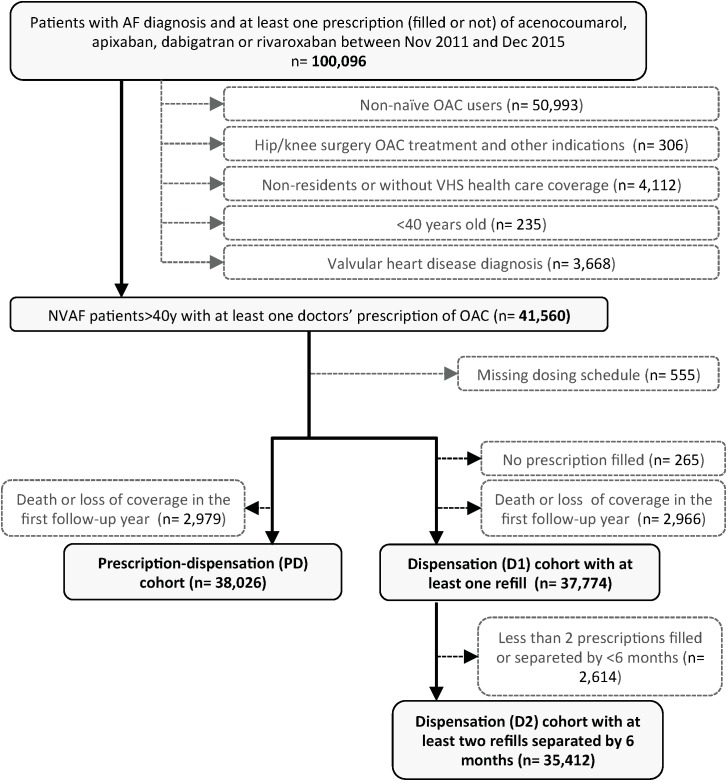
Study flow chart. AF, atrial fibrillation; NVAF, non-valvular atrial fibrillation; OAC, oral anticoagulants; VHA, valencia health agency.

The study was conducted in the population covered by the Valencia Health System (VHS) in Spain. The VHS, part of the Spanish National Health System (sNHS), is funded and mostly provided by the Valencia Government, free at the point of care except for some co-payments for ambulatory medication, and almost universal, covering about 97% of the region’s population (approximately five million inhabitants). We constructed a main cohort comprising all patients with a diagnosis of AF or atrial flutter (ICD-9-CM: 427.31 and 427.32) who started treatment with oral anticoagulants (OACs) (acenocoumarol, apixaban, dabigatran or rivaroxaban) for the prevention of thromboembolic events between November 2011 [date of the market launch of first Non Vitamin K Antagonist Oral Anticoagulant (NOAC)] and December 2015 (Figure 1). The few users of warfarin, phenprocoumon or fluindione, mainly non-residents, were not included.

One year of look-back period was used to define the baseline characteristics of the population and for excluding prevalent OAC users. We defined new users as those patients without any anticoagulant treatment in the 12 months preceding the first doctor’s prescription. We excluded patients with concomitant valvular heart disease (ICD-9-CM: 394.x–397.x, 398.9, 42.4x, V42.2, V43.3, 35.1x, 35.2x), patients with limitations of follow-up (which were people without pharmaceutical coverage by VHS, mainly certain Spanish government employees whose prescriptions are reimbursed by civil servant service insurance mutualities, and thus not included in the electronic records of the VHS), and patients not registered in the municipal census, such as non-residents or temporary residents. Finally, we excluded a small group of patients without information about the prescribed dose because limitations to calculate adherence, and patients who died or lost coverage in the 1st year of follow-up (used to calculate PDC and to classify patients as adherent or non-adherent) because for these patients there was no follow-up available to assess clinical outcomes in the year following their adherence categorization.

### Data Sources

The main source of data was the VHS ambulatory electronic medical record (EMR) that includes information about diagnoses, personal medical history, laboratory test results, lifestyle factors, as well as information about both physician prescriptions and dispensations. The information on hospitalizations was based on the Minimum Basic Dataset (MBDS) at hospital discharge, a synopsis of clinical and administrative information on all hospital discharges, including diagnoses and procedures. The Population Information System (SIP) provides dynamic information on the population covered by the VHS and registers some sociodemographic characteristics, including the geographical/contextual situation of each person and dates and causes of VHS loss of coverage, including death. All these data sources can be linked at an individual level through a unique patient anonymized identifier. A detailed description of source of data is found elsewhere (Sanfélix-Gimeno et al., 2015).

### Adherence Measures

We constructed three cohorts according to the different selection criteria used to calculate alternative forms of PDC (Table 1). The first cohort, called “prescription-dispensation cohort” (PD cohort) corresponds to the formal concept of adherence (“extent to which patients take medications as prescribed by their health care providers”) and estimates days’ supply (the PDC numerator) as days covered by filled medication from the first doctors’ prescription date (index date) to the end of follow-up days provided they are covered by a medical prescription. Days not covered by a medical prescription were censored. Therefore, a patient who has medical prescriptions for 1 year of treatment and does not fill any prescription will have a PDC = 0, while a patient who fills a new treatment for 1 month from the medical prescription and his/her doctor discontinues treatment at the end of that month, will have a PDC of 100% (corresponding to one prescription fully covered with the dispensed treatment).

**Table 1:** Criteria for the construction of adherence and drug exposure measures.

**Prescription-dispensation design with a fixed 12-month time-window (PD Cohort)**	
Prescription fill requirements	No prescription filled is required for inclusion
Index date	Date of first prescription (filled or not)
Numerator (days’ supply)	Days covered by filled medication from the index date to the end of follow-up, provided they are covered by a medical prescription
Denominator (follow-up days)	The 365 days immediately after the index date provided they are covered by a medical prescription
Censoring previous to the end of study date	At the date of death, loss of continuous health plan coverage or periods not covered by a doctor’s prescription during the 12 months of follow-up. No other causes of censoring (including switching or patient discontinuation) are allowed
Immeasurable time	Periods under hospitalization (both acute and long-term care) are not accounted for
Stockpiling	A maximum of 2 months of stockpiling is allowed
**Dispensation-only design with a fixed 12-month time-window (D1 Cohort)**	
Prescription fill requirements	At least one prescription filled is required for inclusion
Index date	Date of first prescription filled
Numerator (days’ supply)	Days covered by filled medication from the index date to the end of follow-up
Denominator (follow days)	The 365 days immediately after the index date
Censoring previous to the end of study date	At the date of death, loss of continuous health plan coverage. No other causes of censoring (including doctors or patient discontinuation or switching) are allowed
Immeasurable time	Periods under hospitalization (both acute and long-term care) are not accounted for
Stockpiling	A maximum 2 months of stockpiling is allowed
**Dispensation-only design with a fixed 12-month time-window, requiring at least two prescriptions filled separated by 6 months (D2 Cohort)**	
Prescription fill requirements	At least two prescription filled separated by 6 months are required for inclusion
Index date	Date of first prescription filled
Numerator (days’ supply)	Days covered by filled medication from the index date to the end of follow-up
Denominator (follow days)	The 365 days immediately after the index date
Censoring previous to the end of study date	At the date of death, lost of continuous health plan coverage. No other causes of censoring (including doctors or patient discontinuation or switching) are allowed
Immeasurable time	Periods under hospitalization (both acute and long-term care) are not accounted for
Stockpiling	A maximum of 2 months stockpiling period is allowed
Rationale	Measure used by the Pharmacy Quality Alliance (PQA). Due to restricted inclusion criteria, the PQA measure excludes most part of non-adherent patients
**Days covered by medication in the 12-month time-window (drug exposure)**	
Fill-up requirements	No prescription filled is required for inclusion
Index date	Date of first prescription (filled or not) for the PD Cohort and date of first prescription filled for D1 and D2 Cohorts.
Numerator (days’ supply)	Days covered by filled medication from the index date to the end of follow-up
Denominator (follow days)	The 365 days immediately after the index date
Censoring previous to the end of study date	No censoring was applied.
Immeasurable time	Periods under hospitalization (both acute and long-term care) are not accounted for
Stockpiling	A maximum 2 months of stockpiling is allowed
Rationale	Rough measure of drug exposure during the follow-up period

The second cohort, called “dispensation cohort with at least one refill” (D1 cohort), represents the most common approach to calculate adherence when information on doctors’ prescription is not available. Patients were required to fill at least one prescription to be included and the index date is the date of this first refill (not the date of the first doctors’ prescription as in the PD cohort). Therefore patients who did not fill any prescription were excluded from this cohort. Days’ supply were estimated as the days covered by filled medication but doctor’s discontinuation of the treatment cannot be captured. Therefore, completely non-adherent patients (who had a PDC = 0 in the PD cohort) were not included in the D1 cohort, while patients whose doctor discontinues the prescription were considered non-compliant (non-adherent). However and despite these inaccuracies regarding the adherence to the medication as prescribed by the doctor, this approach provides a very good proxy of the total patient drug exposure in a period (regardless of whether the doctor maintains the treatment or not and although the period captured may not really correspond to the real beginning of treatment initiation according to the doctors’ first prescription).

The third cohort, called “dispensation cohort with at least two refills” (D2 cohort), corresponds to the definition of OAC adherence used by the Pharmacy Quality Alliance (PQA) (Crivera et al., 2015) that requires at least two prescription refills of OAC with at least 180-days between refills as inclusion criterion for adherence studies, and has been largely used in industry-funded OAC studies (Alberts et al., 2016; McHorney et al., 2016, 2017, 2018; Coleman et al., 2017). Patients with only one refill during the follow-up or who do not have two prescription fills separated by at least 6 months were excluded from the D2 cohort. Therefore this form of PDC estimation excludes by definition patients with short periods of exposure during the follow-up period (or low-adherent patients). As in D1 cohort, days’ supply were calculated as days covered by filled medication from the date of the first refill to the end of the fixed time-window of follow-up.

Summarizing, PD cohort included all patients with an OAC prescription. From those, D1 Cohort included patients who filled at least one prescription, therefore excluding fully non-adherent patients. D2 Cohort included only patients with at least two refills separated by at least 180 days. In the PD cohort, PDC was calculated as the PDC with filled medication out of all days covered by a medical prescription within the 12-month follow-up. In the D1 and D2 cohorts, PDC was calculated as the PDC by filled medication out of the entire duration of the 12-month follow-up regardless of medical prescription coverage.

In all three cohorts, we used a fixed time-window of 12-months from the respective index date as follow-up for adherence assessment (PDC denominator). Cases were censored in case of death or disenrollment, but no other reason for censoring (including switching) was allowed. For the PD cohort, only days under treatment (periods covered with doctors prescriptions) were considered as PDC denominator. Immeasurable time such as hospitalization days were not considered, and stockpiling was allowed for a maximum of 2 months. Days of treatment were always calculated according to doctors’ dosing instructions accompanying the respective prescription (therefore, patients prescribed a daily dose of 110 mg × 2 or 150 mg × 2 who fill a package of 60 tablets, have 30 days of treatment in both cases).

### Outcomes

The pre-specified effectiveness outcomes were mortality for any cause, hospital admission for thromboembolic stroke and hospital admission for gastrointestinal bleeding or intracranial hemorrhage. Only principal discharge diagnoses based on ICD9CM (see Supplementary Table S1 for coding on clinical outcomes) were used to define endpoints. Out-of-hospital mortality was collected from the SIP system and the region’s mortality registry. All outcomes were analyzed separately and only the first event was considered for analysis. Patients were followed from the end of the period used to estimate adherence until the relevant event, health system disenrollment, death, or end of follow-up (Dec 2016 for death, and Dec 2015 for stroke or bleeding), whichever came first. All outcomes were analyzed by intention-to-treat approach, analyzing patients in the group of the first OAC they received (index OAC), regardless of whether a switch to another OAC had occurred during the follow-up.

### Covariates

We considered all available factors potentially related to the risk of thromboembolic events and bleeding. These included demographic and clinical characteristics, and healthcare resource utilization in the preceding 12 months (Table 2). Comorbidity was defined as the presence of an active diagnosis of the particular condition in the EMR within a 12-month period preceding the index date (see Supplementary Table S1 for details on definitions of comorbidities). Concomitant medication (NSAID and antiplatelet) was defined as medication dispensed at least once during the 3-month pre-index period. Based on comorbidity information, concomitant medication and age, we calculated relevant patient-level risk scores of stroke (CHADS2, CHA2DS2-VASC) and bleeding (HAS-BLED). We also included as covariates some specific events (ischemic stroke, bleeding) occurred during the follow-up year used to assess adherence.

**Table 2:** Patient characteristics in the Prescription-Dispensation (PD) cohort and in the Dispensation-only cohorts with at least one (D1) or two (D2) prescriptions filled as inclusion criteria.

	PD Cohort	D1 Cohort	D2 Cohort
*n*	38,026	37,744	35,412
Age; mean (SD)	74.12 (9.85)	74.12 (9.83)	74.35 (9.55)
Female; *n* (%)	18,018 (47.38)	17,891 (47.40)	16,950 (47.87)
**Baseline risk of stroke (CHADS2 and CHA2DS2-VASC) and bleeding (HAS-BLED)**			
CHADS2 score [0–6]; mean (SD)	2.11 (1.25)	2.08 (1.24)	2.11 (1.23)
CHA2DS2-VASC score [0–9]; mean (SD)	3.71 (1.67)	3.66 (1.66)	3.70 (1.64)
HAS BLED score [0–9]; mean (SD)	2.87 (1.18)	2.81 (1.78)	2.83 (1.16)
**Diagnosis**			
Atrial fibrillation; *n* (%)	35,784 (94.10)	35,518 (94.10)	33,387 (94.28)
Atrial flutter; *n* (%)	2,242 (5.90)	2,226 (5.90)	2,025 (5.72)
**Comorbidities and clinical history**			
Hypertension; *n* (%)	30,171 (79.34)	29,608 (78.44)	28,046 (79.20)
Diabetes; *n* (%)	13,070 (34.37)	12,735 (33.74)	12,063 (34.06)
Coronary disease; *n* (%)	6,705 (17.63)	6,437 (17.05)	6,001 (16.95)
Congestive heart failure; *n* (%)	6,166 (16.22)	5,896 (15.62)	5,539 (15.64)
Malignancy; *n* (%)	5,238 (13.77)	5,120 (13.77)	4,795 (13.54)
Previous ischemic stroke or TIA; *n* (%)	5,232 (13.76)	5,064 (13.42)	4,844 (13.68)
Depression; *n* (%)	5,123 (13.47)	4,984 (13.20)	4,721 (13.33)
Renal disease; *n* (%)	4,532 (11.92)	4,392 (11.64)	4,063 (11.47)
Liver disease; *n* (%)	2,761 (7.26)	2,665 (7.06)	2,470 (6.98)
Thromboembolism; *n* (%)	2,624 (6.90)	2,415 (6.40)	2,245 (6.34)
Dementia; *n* (%)	2,876 (7.01)	2,828 (6.95)	2,157 (6.09)
Gastrointestinal bleeding; *n* (%)	1,581 (4.16)	1,483 (3.93)	1,366 (3.86)
Intracranial hemorrhage; *n* (%)	296 (0.78)	288 (0.76)	265 (0.75)
Other bleeding; *n* (%)	8,416 (22.13)	8,012 (21.23)	7,460 (21.07)
**Medication use in the year previous to the index date**			
ASA; *n* (%)	14,456 (38.02)	14,342 (38.00)	13,434 (37.94)
NSAIDs; *n* (%)	7,098 (18.67)	7,046 (18.67)	6,561 (18.53)
Clopidogrel; *n* (%)	1,645 (4.33)	1,628 (4.31)	1,561 (4.41)
ASA + Clopidogrel; *n* (%)	1,568 (4.12)	1,554 (4.12)	1,426 (4.03)
Other antiplatelet drugs; *n* (%)	1,162 (3.06)	1,151 (3.05)	1,094 (3.09)
**Events during the 1st year of follow-up**			
Hospital admission for stroke	337 (0.89)	325 (0.86)	301 (0.85)
Hospital admission for bleeding	409 (1.08)	402 (1.07)	330 (0.93)

*TIA, transient ischemic attack; NSAIDs, non-steroidal anti-inflammatory drugs; ASA, acetylsalicylic acid; PD, prescription-dispensation cohort; D1, dispensation cohort with at least 1 prescription filled; D2, dispensation cohort with at least 2 prescription filled separated by 6 months.*

### Ethics

The study protocol, observational in design and using retrospective anonymized non-identifiable data transferred from the Valencia Ministry of Health to the research team according to the Spanish laws and institutional requirements, was approved by the Ethics Committee for Clinical Research of the General Directorate of Public Health and the Centre for Public Health Research (CEIC DGSP-CSISP, March 5, 2014).

### Statistical Analysis

First, we presented the study population baseline characteristics as means for continuous variables and frequencies for categorical variables for the three cohorts. Second, we estimated crude PDC and PDC80 for each cohort (and for each index drug within each cohort). Third, for each cohort, we described the mean number of days’ supply of medication as a measure of real drug exposure during the 1st year of follow-up (see Table 1 for definitions) for all patients, for adherent patients (PDC ≥ 80) and for non-adherent patients (PDC < 80). In the case of D1 and D2 cohorts, we also estimated the mean number of days’ supply for patients excluded from the respective cohort. Fourth, we compared the classification of patients as adherent or non-adherent among cohorts and also the proportion of patients with ≥80% of the follow-up days covered by medication.

Next, the incidence of death, stroke and hemorrhagic events was described using crude rates per 100 person-years along with 95% CIs separately for each outcome and for each cohort. Finally, we used Cox proportional hazards regression models (adjusted for baseline sociodemographic and clinical characteristics, as well as for non-fatal clinical events occurred during the follow-up period used for calculating adherence) to assess the relationship between adherence (PDC ≥ 80) and health outcomes (death, hospitalization for stroke and hospitalization for bleeding). Patients enrolled from January 2014 (for stroke and bleeding) or January 2015 (for death) were excluded from the Cox models because they did not have a 2nd year of follow-up for the identification of health outcomes.

All statistical analyses were conducted using STATA 14^®^ (StataCorp, College Station, TX), and the 5% level of significance was considered.

## Results

Baseline patient characteristics of the three cohorts were similar (Table 2), not surprisingly as the three cohorts share most of their patients. Mean age was 74 years, slightly less than a half of the patients were women, CHA2DS2-VASC score was 3.7 and HAS-BLED score 2.8. Comorbidities were those expected in this population, with a high prevalence of hypertension (79%), diabetes (34%), coronary disease (17%), heart failure (16%) and previous ischemic stroke or transient ischemic attack (14%). Almost 1% of the patients had one hospital admission for stroke and another 1% for a bleeding episode during the year employed to calculate adherence.

Mean PDC ranged from 96.2% for the PD cohort to 88.5 and 84.7% for D2 and D1 cohorts, respectively (Table 3). The percentage of patients with PDC ≥ 80 ranged from 94.2% (PD cohort), to 79.6% (D2 cohort) and 74.7% (D1 cohort). Patients in the PD cohort had a mean of 306 days covered by medication (74% of the follow-up days; 316 for adherent patients and 149 for non-adherents), compared to 309 days covered in the D1 cohort (85% of the follow-up days; adherent patients: 352; non-adherent patients: 181) and to 323 in the D2 cohort (88% of the follow-up days; adherents: 352; non-adherents: 209). The 295 patients excluded from cohort D1 with respect to the PD cohort (patients who did not refill any prescription during the year after the first doctors’ prescription) had a mean of 41 covered days (most part of patients were fully incompliant but some patients restarted after a year from the first prescription), and the 4,706 patients excluded from cohort D2 (patients with less than two refills or less than 180 days between refills) had a mean of 89 covered days.

**Table 3:** Proportion of days covered and days’ supply during 1 year of follow-up, according to prescription-dispensation and dispensation-only based designs.

	Acenocoum.	Apixaban	Dabigatran	Rivaroxaban	Total OAC
**Prescription-dispensation cohort (patients with at least one prescription; PD Cohort)**					
*n*	29,574	2,075	3,197	3,180	38,026
PDC; mean (95% CI)	96.39 (96.26–96.52)	95.57 (94.97–96.17)	95.87 (95.40–96.34)	94.65 (94.07–95.23)	96.16 (96.03–96.28)
Patients with PDC ≥ 80; % (95% CI)	94.46 (94.19–94.71)	93.59 (92.45–94.57)	93.49 (92.58–94.30)	92.52 (91.55–93.38)	94.17 (93.93–94.40)
Days’ supply; mean (95% CI)	305.9 (304.9–306.9)	322.8 (319.1–326.5)	297.9 (294.3–301.5)	307.7 (304.2–311.3)	306.3 (305.4–307.2)
Days’ supply if PDC ≥ 80%; mean (95% CI)	314.6 (313.6–315.5)	335.6 (332.4–338.7)	309.8 (306.5–313.2)	323.6 (320.6–326.7)	316.0 (315.2–316.7)
Days’ supply if PDC < 80%; mean (95% CI)	158.6 (154.3–162.8)	136.7 (120.7–152.6)	126.0 (113.8–138.1)	111.1 (98.57–123.7)	149.1 (145.3–152.9)
**Dispensation-only cohort (patients with at least one dispensation; D1 cohort)**					
*n*	29,411	2,047	3,162	3,124	37,744
PDC (mean, 95% CI)	84.37 (84.10–84.63)	89.59 (88.62–90.56)	82.72 (81.77–83.67)	86.42 (85.51–87.33)	84.68 (84.44–84.92)
Patients with PDC ≥ 80; % (95% CI)	73.44 (72.93–73.94)	84.86 (82.23–86.34)	74.07 (72.51–75.57)	80.60 (79.18–81.95)	74.70 (74.26–75.13)
Days’ supply; mean (95% CI)	307.8 (306.9–308.8)	326.7 (323.1–330.2)	301.8 (298.3–305.3)	315.1 (311.7–318.4)	308.9 (308.1–309.8)
Days’ supply if PDC ≥ 80%; mean (95% CI)	351.2 (350.9–351.5)	357.2 (356.4–358.1)	354.1 (353.4–354.9)	356.4 (355.7–357.2)	352.3 (352.0–352.5)
Days’ supply if PDC < 80%; mean (95% CI)	187.9 (186.2–187.7)	155.3 (145.6–165.0)	152.3 (146.4–158.1)	143.1 (136.0–150.3)	181.0 (179.3–182.6)
Days’ supply for patients excluded from D1 cohort; mean (95% CI)^∗^	24.3 (12.7–35.9)	80.3 (31.8–128.8)	78.5 (32.9–124.0)	48.9 (19.1–78.8)	41.5 (29.9–53.1)
**Dispensation-only cohort (patients with at least two dispensations separated by 6 months; D2 cohort)**					
*n*	27,773	1,923	2,848	2,868	35,412
PDC; mean (95% CI)	87.74 (87.52–87.97)	93.85 (93.20–94.50)	89.05 (88.32–89.79)	92.06 91.40–92.71	88.53 (88.34–88.73)
Patients with PDC ≥ 80; % (95% CI)	77.75 (77.25–78.23)	90.22 (88.81–91.47)	82.19 (80.75–83.56)	87.69 (86.44–88.84)	79.59 (79.17–80.00)
Days’ supply; mean (95% CI)	320.2 (319.4–321.0)	342.4 (340.1–344.8)	325.0 (322.3–327.7)	335.9 (333.5–338.3)	323.1 (322.4–323.8)
Days’ supply if PDC ≥ 80%; mean (95% CI)	351.2 (351.0–351.5)	357.5 (356.8–358.3)	354.2 (353.5–354.9)	356.7 (356.1–357.4)	352.4 (352.1–352.6)
Days’ supply if PDC < 80%; mean (95% CI)	211.8 (210.2–213.5)	203.1 (193.0–213.2)	190.2 (183.3–197.1)	187.3 (178.4–196.1)	208.9 (207.3–210.5)
Days’ supply for patients excluded from D2 cohort; mean (95% CI)^∗∗^	92.1 (89.1–95.1)	82.8 (71.4–94.2)	90.7 (83.8–97.6)	76.8 (69.1–84.5)	89.5 (87.0–92.1)

*PDC, proportion of days covered; CI, confidence interval; PDC80, % of patients with PDC values equal or above 80%. ^∗^282 patients with a prescription excluded from D1 cohort. ^∗∗^2614 patients that did not filled at least two prescription separated by 6 months excluded from D2 cohort.*

When observing specific drugs (Table 3), differences in mean PDC—although significant in some cases—were negligible in the PD cohort (from 96% for acenocoumarol to 94% for rivaroxaban), but widened in the dispensation-based cohorts (from 89% for apixaban to 83% for dabigatran in the D1 cohort and from 94% for apixaban to 88% for acenocoumarol in the D2 cohort). Regarding patients with PDC ≥ 80%, all drugs showed high adherence figures (93–94%) in the PD cohort. In the D1 cohort, these figures declined notably for acenocoumarol and dabigatran (73–74% of patients with PDC ≥ 80%) and to a lesser extent in the case of rivaroxaban (81%) and apixaban (84%). The D2 cohort showed even greater differences between drugs: from 90% of patients with PDC ≥ 80% when apixaban was the index drug (or 88% for rivaroxaban) to 82% for dabigatran or 78% for acenocoumarol. Therefore, while in the PD cohort acenocoumarol showed the highest number of patients with PDC equal to or greater than 80% (95%), in both D1 and D2 cohorts, rivaroxaban and especially apixaban showed the best adherence results, with acenocoumarol falling to 78% (D2 design) or 73% (D1 design).

A 20% of patients classified as adherent (PDC ≥ 80%) in the PD cohort had less than 80% days covered by medication, by virtually none in cohorts D1 and D2 whose classification of “adherence” is very close to the actual exposure to OACs (Table 4). D1 and D2 cohorts offer a very similar adherence classification, even at the expense of excluding a significant proportion of non-adherent patients in the case of the D2 cohort.

**Table 4:** Drug exposure (days’ supply) and classification of patients as adherent (PDC ≥ 80%) or non-adherent (PDC < 80%), according to different approaches to calculate PDC.

		PD Cohort			D1 Cohort			D2 Cohort		
		PDC < 80	PDC ≥ 80	Total	PDC < 80	PDC ≥ 80	Total	PDC < 80	PDC ≥ 80	Total
Days’ supply	<80%	2,218	7,719		9,572	40		7,236	21	
		(5.8%)	(20.3%)		(25.3%)	(0.1%)		(20.4%)	(0.1%)	
	≥80%	0	28,089		0	28,202		0	28,200	
		(0.0%)	(73.9%)		(0.0%)	(74.6%)		(0.0%)	(79.5%)	
	Total	2,218 (5.8%)	35,808 (94.2%)	38,026	9,572 (25.3%)	28,242 (74.7%)	37,814	7,236 (20.4%)	28,221 (79.6%)	35,457
PD cohort	PDC < 80				1,909	98		1,396	89	
					(5.0%)	(0.3%)		(3.9%)	(0.3%)	
	PDC ≥ 80				7,663 (20.3%)	28,144 (74.4%)		5,840 (16.5%)	28,132 (79.3%)	
	Total				9,572 (25.3%)	28,242 (74.7%)	37,814	7,236 (20.4%)	28,221 (79.6%)	35,457
D1 cohort	PDC < 80							7,236 (20.4%)	0 (0.0%)	
	PDC ≥ 80							0 (0.0%)	28,221 (79.5%)	
	Total							7,236 (20.4%)	28,221 (79.5%)	35,457

*PDC, proportion of days covered; PDC80, percentage of patients with PDC ≥ 80%; PD, prescription-dispensation cohort; D1, dispensation-only cohort with at least 1 refill; D2, dispensation-only cohort with at least two refills. Shaded cells indicate discrepancies in adherence classification according to different approaches for PDC estimation.*

Crude events rate by 100 person-years during follow-up (1.9 years for mortality and 1.4 for stroke and bleeding) were about 6.7 for death, 0.9 for stroke and 1.4 for bleeding (Table 5). Unadjusted proportional hazard regression showed a significant protective effect of adherence on mortality (Hazard Ratio [HR] between 0.79 and 0.93 depending on the cohort) and also on stroke in the D1 and D2 cohorts (HR of 0.62 and 0.69, respectively) but not in the PD cohort, with no effect on admission for bleeding episodes. Multivariate proportional hazard regression models (Table 5 and Supplementary Tables S2–S4) showed similar results, with adherence associated with a significant reduced risk of death for the three cohorts (adjusted HR between 0.80 and 0.86). Stroke risk reduction was significant for D1 and D2 cohorts (adjusted HR between 0.61 and 0.64), and very close to statistical significance for the PD cohort (adjusted HR 0.66, 95CI: 0.43–1.02). Adherence had no effect on bleeding.

**Table 5:** Crude health outcomes rates and association between adherence (PDC ≥ 80%) and health outcomes according to different approaches for PDC estimation (crude and adjusted hazard ratios).

	Crude incidence rates per 100 person-years		Hazard ratios (95% CI)	
	Adherent	Non-adherent	Unadjusted	Adjusted
**PD cohort**				
Death (any cause)	6.61 (6.42–6.81)	8.24 (7.32–9.28)	0.79 (0.79–0.90)	0.82 (0.72–0.93)
Ischemic stroke	0.88 (0.79–0.98)	1.28 (0.84–1.97)	0.68 (0.44–1.05)	0.66 (0.43–1.02)
Bleeding	1.45 (1.34–1.57)	1.34 (0.88–2.04)	1.09 (0.71–1.67)	1.04 (0.68–1.58)
**D1 cohort**				
Death (any cause)	6.38 (6.16–6.60)	7.46 (7.08–7.86)	0.86 (0.81–0.91)	0.80 (0.75–0.86)
Ischemic stroke	0.79 (0.70–0.90)	1.16 (0.98–1.38)	0.69 (0.56–0.85)	0.64 (0.51–0.79)
Bleeding	1.45 (1.32–1.60)	1.45 (1.24–1.69)	1.00 (0.84–1.20)	0.96 (0.79–1.15)
**D2 cohort**				
Death (any cause)	6.38 (6.16–6.60)	6.94 (6.53–7.37)	0.93 (0.86–0.99)	0.86 (0.80–0.93)
Ischemic stroke	0.80 (0.70–0.90)	1.20 (1.00–1.46)	0.66 (0.53–0.83)	0.61 (0.49–0.77)
Bleeding	1.45 (1.32–1.60)	1.59 (1.35–1.87)	0.91 (0.76–1.10)	0.86 (0.71–1.04)

*PD, prescription-dispensation cohort; D1, dispensation-only cohort with at least 1 refill; D2, dispensation-only cohort with at least two refills. n: 38,026 (PD cohort), 37,744 (D1 cohort) and 35,412 (D2 cohort) for death, and 28,339 (PD cohort), 28,128 (D1 cohort) and 26,451 (D2 cohort) for stroke and bleeding. Mean follow-up time: 1.86 years for death and 1.44 years for stroke and bleeding.*

## Discussion

Our study highlights the importance of patient adherence to OAC medications. Irrespective of the approach used for measuring PDC, adherence to OAC in NVAF patients was associated with a substantial reduction in mortality and (except in the PD cohort) in the incidence of thromboembolic stroke, without a significant increase in admissions for hemorrhagic episodes. Significance of stroke risk reduction in the PD cohort was probably not achieved because a substantial number of patients with low drug exposure due that physician-initiated treatment discontinuation (20% of patients classified as adherent, with PDC ≥ 80%, had less than 80% of days of exposure) were classified as adherent.

The association between high levels of medication adherence and better outcomes is, in general, consistent with previous research (all with D1 or D2 designs) (Shore et al., 2014; Alberts et al., 2016; Yao et al., 2016; Borne et al., 2017; Deshpande et al., 2018) evaluating the association between adherence to OACs and health outcomes using the PDC80 threshold. This consistency, despite methodological differences (some of the referenced publications include experienced users, censor switchers or censor follow-up at the time of the last refill, use variable time-windows and other process differences), also strengthens the importance of adherence to approximate the real-life OAC benefits to those obtained in clinical trials.

To the best of our knowledge our study is the first using a prescription-based design to assess OAC adherence (and of the very few who have used this methodology in any other condition). This specific design evidences that patients (at least in OAC treatment in the Valencia region public healthcare setting) are highly adherent to medical recommendations, with 94% of patients above PDC80 when adherence is assessed against doctors’ prescription. Nevertheless, and due to doctor’s discontinuation, this high “adherence” translates into a mean of only 74% of patients with at least 80% of the follow-up days covered by medication. This finding suggests that physician-initiated treatment discontinuation is a major contributor to reduced OAC exposure. When using the dispensation-based D1 design “adherence” figures represent medication exposure more accurately (PDC80: 75%; days’ supply: 74%), but at the expense of misestimating “real adherence” to the medication prescribed, attributing to patients a supposed loss of adherence when it is actually treatment discontinuation by doctors. This finding is of especial relevance because most interventions aimed to improve medication adherence are focused on patient’s behavior. In the light of our results, and especially for essential treatments and in some contexts the appropriateness of medical discontinuation should be also considered when tackling the issue of real-world drug adherence. Moreover, dispensation-only designs to assess adherence classifies some adherent patients as non-adherent, and thus, targeting improvement interventions to these patients would be inefficient.

Adherence to medical recommendations was high and similar for the most used VKA in Spain (acenocoumarol, PDC80 = 94%) and any of the marketed NOAC (PDC80 between 92 and 94%), contrasting the worst adherence to NOAC (vs. VKA) speculated by some authors based on the lack of close monitoring (INR control) (Rodriguez et al., 2013; Hendriks et al., 2017; Burn and Pirmohamed, 2018). In the same way, adherence to VKA—always taking the medical prescription as reference—does not seem inferior to NOAC adherence, contrary to what is suggested by studies assessing the comparative adherence to VKA and NOAC (McHorney et al., 2017). However, doctors discontinue treatment to a greater extent in patients initiating with acenocoumarol (PDC80 in D1 Cohort: 73.4%) or dabigatran (PDC80 in D1 cohort: 74.1%) than rivaroxaban or apixaban (PDC80: 80.6 and 84.9% in the D1 cohort). This may partially explain why in the dispensation-only cohorts used by several adherence comparative studies the latter medications appear to have better adherence results than VKA or dabigatran (Crivera et al., 2015; Hanemaaijer et al., 2015; McHorney et al., 2015, 2017; Alberts et al., 2016; Beyer-Westendorf et al., 2016; Brown et al., 2016, 2017; Coleman et al., 2016; Yao et al., 2016; Borne et al., 2017; Manzoor et al., 2017; Maura et al., 2017; Mueller et al., 2017). Note that these differences are not due to switching to another OAC (an aspect not assessed in our study, in which, by design, the days’ supply of the new OAC after switching would continue to be considered in the index drug) but to discontinuation of treatment without restart during the follow-up period.

Finally, our study showed that adherent patients (defined as patients above PDC80) and non-adherent patients have different medication exposure depending on the method employed to calculate PDC (with mean days’ supply ranging from 10 to 12 months for adherent patients and from 5 to 7 months for non-adherent patients, depending on the cohort). Also, we showed that, in D1 and D2 cohorts, the design fails to include relevant groups of low-adherent patients as non-adherent patients or patients with only one refill, and also periods of non-adherence (as primary or early non-adherence) are ignored (Rodriguez-Bernal et al., 2018). Classification of patients as adherent or non-adherent based in the PDC80 threshold is sensitive to the method used for calculating PDC, and the assessment of the relationship between adherence and health outcomes compare different groups regarding drug exposure (e.g., in some designs outcomes are compared between patients with 10 vs. 4.6 months of average treatment, while in others designs outcomes are compared between patients with 12 vs. 7 months of average treatment). These results emphasize the need to standardize study designs and methods of calculation of days’ supply-based adherence measures with refill databases.

In this regard, the PQA method to estimate adherence requiring two prescriptions filled by at least 180 separate days excludes a relevant part of non-adherent patients artificially improving adherence with respect to the D1 design and without any additional advantage over this method. In fact, some studies linking doctor’s prescription to pharmacy dispensation show that up to one in three patients either never fill their initial prescription or discontinue the drug after a single fill (Fischer et al., 2010; Shrank et al., 2010; Raebel et al., 2012).

### Limitations

Our work is subject to several limitations. First, we used prescription and dispensing data to measure adherence, but patients do not necessarily consume all the drugs filled. Nevertheless, several studies have shown a high consistency between dispensation and patient consumption (Grymonpre et al., 1998; Grymonpre et al., 2006; Márquez-Contreras et al., 2018; Mehta et al., 2018).

Second, we used information on prescribing dosing schedule to construct days’ supply. This method is accurate to estimate exposure to NOAC, but may be more imprecise for acenocoumarol (dosing is frequently modified after INR control visits and may not be registered in the prescription). Since acenocoumarol is the most prevalent OAC in Spain (García-Sempere et al., 2017), this greater imprecision could affect—to some extent—some study results. Even so, we believe that this method is more accurate than using average doses or similar methods used in other studies.

Third, the high adherence to doctors’ prescription found in our study may not be generalizable to other conditions and contexts. Despite the increase of co-payments as part of the measures to reduce public spending in July 2012 (González López-Valcárcel et al., 2017), the level of co-payment for chronic disease medications and for retired people in the Spanish NHS is low. In this sense, co-payment may be a weaker barrier to adherence in Spain than in countries with higher patient cost-sharing (Choudhry et al., 2007, 2011, 2014).

Fourth, NOAC drugs have been marketed at different moments in time. It can be assumed that patterns of initiation with a specific drug when five alternative treatments are available may be different from those when less options were on the market. We carried out a sensibility analysis calculating PDC80 only for patients initiating in the period where all five alternatives were marketed (the period after apixaban release, Supplementary Table S5). No relevant changes were found with regard to our main analysis.

Fifth, regarding the relationship between adherence and outcomes, we cannot rule out the presence of a healthy adherer effect, a type of bias reflecting patient behavior, not easily identifiable and quantifiable, that could positively affect health outcomes, being the benefits incorrectly attributed to medication adherence (Chewning, 2006; Shrank et al., 2011; Ladova et al., 2014). In the same way, and despite the multivariate adjustment made, the possibility of reverse causation in our analysis between adherence and outcomes cannot be ruled out: poor expected outcomes could lead to discontinuation of treatment (by the doctor or the patient) being the outcome wrongly attributed to the discontinuation. Addressing these limitations requires the use of complex study designs and modeling techniques to control confounding by indication (patients who discontinue are different from patient who not) and information biases, both being issues that have barely been addressed to date in studies linking drug adherence and outcomes (Yu et al., 2010; LaFleur et al., 2011; Nelson et al., 2013).

## Conclusion

Studies assessing adherence with data from refill databases and using a 80% threshold on days’ supply measures tend to exclude low-adherent patients and periods of non-adherence and, also, misclassify periods of treatment discontinuation by doctors as patient non-adherence. Designs based on doctors’ prescription solve these problems, but require additional information about the actual exposure to treatment since these designs classify as adherent (to the medical prescription) patients with very low drug exposure whose doctors have discontinued treatment, a major reason for low OAC exposure.

The 80% threshold to classify patients as adherent or non-adherent can include very different groups of patients according to the methods used for the construction of days’ supply measures, highlighting the need to standardize (and transparently detail) the procedures used for the construction of these measures. In any case, high adherence and high exposure to evidence-based treatments, such as OAC in NVAF patients, seem to be associated with better outcomes, strengthening the need to develop strategies (focused on the patient, doctors and/or health organizations) to bring real-world outcomes closer to those of the clinical trials.

## Author Contributions

GS-G, AG-S, and SP conceived the study and wrote the first draft of the manuscript. GS-G and SP are guarantors and take full responsibility for the integrity of the data and the accuracy of the data analysis. IH-N, AG-S, CR-B, YS-A, SP, and GS-G participated in the study design and methodology. IH-N and SP performed the analysis. All authors participated in interpretation of data, and contributed to the critical revision of the manuscript for important intellectual content. All authors agree to be accountable for all aspects of the work and have read and approved the final manuscript.

## Conflict of Interest Statement

The FISABIO foundation (a non-for-profit research institution depending on the Valencia Ministry of Health) had a Collaboration Agreement with Boehringer Ingelheim to conduct non-conditioned independent research in chronic health care, pharmacoepidemiology and medical practice variation (2013–2014), and received an unrestricted research grant from Daiichi-Sankyo (2017), both not related with the current study. GS-G participated in 2014 in an advisory meeting of Boehringer Ingelheim. None of the mentioned institutions/firms were involved in the design and conduct of the study; the collection, management, analysis, and interpretation of the data; or the preparation, review or approval of the manuscript, or in the decision to submit it for publication. The views presented here are those of the authors and not necessarily those of the FISABIO Foundation, the Valencia Ministry of Health or any other institution.
