# Supplementary material for: Estimating Adherence Based on Prescription or Dispensation Information: Impact on Thresholds and Outcomes. A Real-World Study With Atrial Fibrillation Patients Treated With Oral Anticoagulants in Spain
Source: Front Pharmacol. 2018 Dec 3;9:1353. doi: 10.3389/fphar.2018.01353 (PMC6287024; doi:10.3389/fphar.2018.01353)
Supplement: Supplementary file 1 [file Table_1.DOC]

| **Table S1. International Classification of Disease, 9th edition, Clinical Modification (ICD-9-CM) codes used to define study clinical outcomes and comorbidities.** | |
| --- | --- |
| ***Clinical Outcomes*** | |
| Isquemic stroke | 433.x1, 434.x1, 436.xx |
| TIA | 435.xx |
| GI bleeding | 455.2, 455.5, 455.8, 456.0, 456.20, 459.0,530.7, 530.82, 531.00, 531.01, 531.20, 531.21, 531.40, 531.41, 531.60, 531.61, 533.00, 533.01, 533.20, 533.21, 533.40, 533.41, 533.61, 534.00, 534.01, 534.20, 534.21, 534.40, 534.41, 534.61, 535.01, 535.11, 535.21, 535.31, 535.41, 535.51, 535.61, 537.83, 562.02, 562.03, 562.12, 562.13, 568.81, 569.3, 569.85, 578.0, 578.1, 578.9 |
| Major GI bleeding | GI bleeding + ICD-9 procedure code of blood or blood components transfusion (99.03, 99.04, 99.05, 99.06, 99.07, 99.09) |
| Intracranial haemorrhage | 430.xx, 431.xx, 432.xx, 852.0x, 852.2x, 852.4x, 853.0x |
|  | |
| ***Comorbidities*** | |
| Congestive heart failure | 428.xx, 398.91, 402.01, 402.11, 402.91, 404.01, 404.11, 404.03, 404.13, 404.91, 404.93 |
| Hypertension | 401.xx, 402.xx, 403.xx, 404.xx, 405.xx, 437.2 |
| Diabetes | 250.xx, 357.2, 362.0x, 366.41 |
| Liver disease | 571.5, 572.0, 572.1, 572.2, 572.3, 572.4, 572.8, 789.5, 789.59, 570, 571.6, 571.8, 571.9, 573.0, 573.4, 573.8, 573.9, 782.4, 789.1, 790.4, 790.5, 794.8, V42.7 |
| Renal disease | 584.5, 584.6, 584.7, 584.8, 584.9, 586, 585, 585.3, 585.4, 585.5, 585.6, 585.9, 792.5, V42.0, V45.1, V54.11, V45.12, V56.0, V56.1, V56.2, V56.31, V56.32, V56.8 |
| Previous ischemic stroke or TIA | 433.x1, 434.x1, 435.x, 436, 438.x |
| Coronary artery disease | 410.xx-414.xx |
| Deep vein thromboembo-lism & pulmonary embolism | 451.11, 451.19, 451.2, 451.81, 451.9, 453.40, 453.41, 453.42, 453.5x, 453.8, 453.9, 415.1, 415.11, 415.13, 415.19 |
| Hemorrhagic stroke | 430.xx, 431.xx, 432.x, 852.0x, 852.2x, 852.4x, 853.0x |
| Gastrointestinal bleeding | 455.2, 455.5, 455.8, 456.0, 456.20, 459.0, 530.7, 530.82, 531.00, 531.01, 531.20, 531.21, 531.40, 531.41, 531.60, 531.61, 533.00, 533.01, 533.20, 533.21, 533.40, 533.41, 533.61, 534.00, 534.01, 534.20, 534.21, 534.40, 534.41, 534.61, 535.01, 535.11, 535.21, 535.31, 535.41, 535.51, 535.61, 537.83, 562.02, 562.03, 562.12, 562.13, 568.81, 569.3, 569.85, 578, 578.0, 578.1, 578.9 |
| Other major bleeding | 423.0, 459.0, 593.81, 719.1, 719.10, 719.11, 719.12, 719.13, 719.14, 719.15, 719.16, 719.17, 719.18, 719.19, 784.7, 784.8, 786.3, 599.7, 285.1, 280.9, 280.0, 285.9 |
| Bleeding history or predisposition | 430.xx, 431.xx, 432.x, 852.0x, 852.2x, 852.4x, 853.0x, 455.2, 455.5, 455.8, 456.0, 456.20, 459.0, 530.7, 530.82, 531.00, 531.01, 531.20, 531.21, 531.40, 531.41, 531.60, 531.61, 533.00, 533.01, 533.20, 533.21, 533.40, 533.41, 533.61, 534.00, 534.01, 534.20, 534.21, 534.40, 534.41, 534.61, 535.01, 535.11, 535.21, 535.31, 535.41, 535.51, 535.61, 537.83, 562.02, 562.03, 562.12, 562.13, 568.81, 569.3, 569.85, 578, 578.0, 578.1, 578.9, 423.0, 459.0, 593.81, 719.1, 719.10, 719.11, 719.12, 719.13, 719.14, 719.15, 719.16, 719.17, 719.18, 719.19, 784.7, 784.8, 786.3, 599.7, 285.1, 280.9, 280.0, 285.9 |
| Malignancy | 140, 141, 142, 143, 144, 145,146, 147, 148, 149, 150, 151, 152, 153, 154, 155, 156, 157, 158, 159, 160, 161, 162, 163, 164, 165, 166, 167, 168, 169, 170, 171, 172, 173, 174, 175, 176, 177, 178, 179, 180, 181, 182, 183, 184, 185, 186, 187, 188, 189, 190, 191, 192, 193, 194, 195, 196, 197, 198, 199, 200, 201, 202, 203, 204, 205, 206, 207, 208, 209, 210, 211, 212, 213, 214, 215, 216, 217, 218, 219, 220, 221, 222, 223, 224, 225, 226, 227, 228, 229, 230, 231, 232, 233, 234, 235, 236, 237, 238, 239. |
| Depression | 296.2, 296.3, 298.0, 300.4, 301.12, 311. |
| Dementia | 290, 294, 330, 331. |
| TIA: Transient Ischemic Attack; ICD-9-CM: International Classification of Diseases, 9th Revision, Clinical Modification; GI: gastrointestinal | |

| **Table S2. Association between adherence (PDC≥80%) and death during the follow-up according to the different approaches to estimate PDC. Cox proportional hazards regression models** | | | |
| --- | --- | --- | --- |
|  | **PD Cohort**  **HR (95%CI)** | **D1 Cohort**  **HR (95%CI)** | **D2 Cohort**  **HR (95%CI)** |
| ***Adherence*** | | | |
| PDC ≥80% (vs. PDC<80%) | **0.82 (0.72 – 0.93)** | **0.80 (0.75 – 0.86)** | **0.86 (0.80 – 0.93)** |
| ***Sociodemographic characteristics*** | | | |
| Women (vs. Men) | **0.70 (0.66 – 0.74)** | **0.70 (0.66 – 0.75)** | **0.69 (0.65 – 0.74)** |
| Age 65-74y (vs. <65y) | **1.67 (1.41 – 1.97)** | **1.68 (1.42 – 1.99)** | **1.65 (1.38 – 1.97)** |
| Age 75-84y (vs. <65y) | **3.76 (3.20 -4.43)** | **3.81 (3.23 – 4.49)** | **3.83 (3.22 – 4.56)** |
| Age 85+ (vs. <65y) | **7.48 (6.31 – 8.87)** | **7.59 (6.39 – 9.02)** | **7.67 (6.40 – 9.19)** |
| ***Clinical characteristics and comorbidities at baseline*** | | | |
| Atrial fibrillation (vs. Atrial flutter) | **1.35 (1.16 – 1.56)** | **1.37 (1.18 – 1.59)** | **1.43 (1.22 – 1.68)** |
| CHA2DS2-VASC moderate risk (vs. low) | 1.00 (0.65 – 1.54) | 1.20 (0.78 – 1.83) | 1.10 (0.70 – 1.75) |
| CHA2DS2-VASC high risk (vs. low) | 1.42 (0.95 – 2.11) | **1.53 (1.03 – 2.28)** | 1.37 (0.89 – 2.11) |
| HAS BLED ≥3 (vs. <3) | 0.95 (0.87 – 1.03) | 0.97 (0.89 – 1.06) | 0.95 (0.87 – 1.04) |
| Heart failure | **1.75 (1.64 – 1.88)** | **1.78 (1.66 – 1.90)** | **1.80 (1.67 – 1.93)** |
| Diabetes | **1.24 (1.16 – 1.31)** | **1.25 (1.18 – 1.33)** | **1.26 (1.18 – 1.34)** |
| Renal disease | **1.45 (1.34 – 1.56)** | **1.43 (1.32 – 1.55)** | **1.44 (1.32 – 1.56)** |
| Hypertension | 1.02 (0.94 – 1.22) | 1.03 (0.94 – 1.12) | 1.04 (0.95 – 1.14 |
| Liver disease | 1.04 (0.93 – 1.77) | 1.05 (0.93 – 1.19) | 1.04 (0.92 – 1.18) |
| Malignancy | **1.29 (1.20 – 1.40)** | **1.28 (1.18 – 1.38)** | **1.24 (1.17 – 1.37)** |
| Dementia | **1.76 (1.60 – 1.93)** | **1.75 (1.59 – 1.93)** | **1.82 (1.65 – 2.01)** |
| Depression | 1.07 (0.98 – 1.17) | 1.08 (0.99 – 1.18) | **1.10 (1.01 – 1.21)** |
| Previous stroke | **1.28 (1.18 – 1.38)** | **1.26 (1.17 – 1.37)** | **1.27 (1.17 – 1.37)** |
| Previous acute coronary syndrome | **1.21 (1.13 – 1.30)** | **1.21 (1.12 – 1.30)** | **1.21 (1.17 – 1.37)** |
| Previous embolism | **1.27 (1.15 – 1.40)** | **1.21 (1.09 – 1.35)** | **1.24 (1.12 – 1.38)** |
| Previous haemorrhagic stroke | 1.07 (0.79 – 1.46) | 1.07 (0.78 – 1.48) | 1.11 (0.80 – 1.55) |
| Previous GI bleeding | 0.95 (0.83 – 1.09) | 0.98 (0.86 – 1.38) | 1.02 (0.88 – 1.18) |
| Previous other bleeding | **1.24 (1.16 – 1.33)** | **1.22 (1.13 – 1.30)** | **1.21 (1.12 – 1.30)** |
| ***Events in the first year of follow-up*** |  |  |  |
| Ischemic stroke | **1.82 (1.44 – 2.29)** | **1.74 (1.37 – 2.21)** | **1.67 (1.29 – 2.15)** |
| Hemorrhagic stroke | **1.60 (1.08 – 2.38)** | **1.54 (1.04 – 2.29)** | **1.59 (1.06 – 2.41)** |
| Gastrointestinal bleeding | 1.07 (0.74 – 1.55) | 0.97 (0.66 – 1.42) | 0.83 (0.53 – 1.30) |
| Major gastrointestinal bleeding | **1.57 (1.01 – 2.45)** | 1.49 (0.95 – 2.33) | **1.78 (1.09 – 2.90)** |
|  |  |  |  |
| ***Model information*** | | | |
| *n* | 38,026 | 37,744 | 35,412 |
| *Deaths* | 4,737 | 4,687 | 4,317 |
| *Person-years, n* | 70,830 | 70,275 | 66,384 |
| *Mean follow-up, years* | 1,86 | 1,86 | 1,87 |
| *Rate, 100 person/years (95%CI)* | 6.69 (6.50 – 6.88) | 6.67 (6.48 – 6.86) | 6.50 (6.31 – 6.69) |
| *Harrell’s C Statistic* | 0.730 | 0.730 | 0.731 |
| PDC: Proportion of days covered; PDC80: Percentage of patients with PDC ≥ 80%. PD: Prescription-dispensation cohort; D1: Dispensation-only cohort with at least 1 refill; D2: Dispensation-only cohort with at least two refills. HR: Hazard Ratio; | | | |

| **Table S3. Association between adherence (PDC≥80%) and hospital admission for stroke during the follow-up, according to the different approaches to estimate PDC. Cox proportional hazards regression models** | | | |
| --- | --- | --- | --- |
|  | **PD Cohort**  **HR (95%CI)** | **D1 Cohort**  **HR (95%CI)** | **D2 Cohort**  **HR (95%CI)** |
| ***Adherence*** | | | |
| PDC ≥80% (vs. PDC<80%) | 0.66 (0.43 – 1.02) | **0.64 (0.51 – 0.79)** | **0.62 (0.49 – 0.78)** |
| ***Sociodemographic characteristics*** | | | |
| Women (vs. Men) | 0.94 (0.76 – 1.17) | 0.93 (0.75 – 1.16) | 0.91 (0.72 – 1.14) |
| Age 65-74y (vs. <65y) | 1.29 (0.81 – 2.06) | 1.35 (0.84 – 2.17) | 1.37 (0.84 – 2.24) |
| Age 75-84y (vs. <65y) | **2.03 (1.29 – 3.18)** | **2.09 (1.32 – 3.32)** | **2.08 (1.30 – 3.36)** |
| Age 85+ (vs. <65y) | **2.62 (1.59 – 4.31)** | **2.51 (1.50 – 4.22)** | **2.42 (1.41 – 4.16)** |
| ***Clinical characteristics and comorbidities at baseline*** | | | |
| Atrial fibrillation (vs. Atrial flutter) | **1.94 (1.03 – 3.65)** | **1.97 (1.05 – 3.70)** | **2.60 (1.22 – 5.51)** |
| CHA2DS2-VASC moderate risk (vs. low) | **0.31 (0.11 – 0.89)** | **0.20 (0.06 – 0.64)** | **0.19 (0.06 – 0.61)** |
| CHA2DS2-VASC high risk (vs. low) | 0.52 (0.23 – 1.19) | 0.55 (0.24 – 1.24) | 0.49 (0.20 – 1.13) |
| HAS BLED ≥3 (vs. <3) | 1.22 (0.90 – 1.65) | 1.16 (0.86 – 1.57) | 1.10 (0.81 – 1.50) |
| Heart failure | 1.11 (0.84 – 1.46) | 1.06 (0.80 – 1.40) | 1.04 (0.77 – 1.39) |
| Diabetes | **1.26 (1.02 – 1.56)** | **1.32 (1.06 – 1.64)** | **1.32 (1.06 – 1.65)** |
| Renal disease | 0.86 (0.62 – 1.21) | 0.90 (0.64 – 1.26) | 0.93 (0.65 – 1.32) |
| Hypertension | 1.18 (0.85 – 1.65) | 1.20 (0.86 – 1.67) | 1.24 (0.88 – 1.73) |
| Liver disease | 1.06 (0.70 – 1.63) | 1.11 (0.73 – 1.69) | 1.21 (0.79 – 1.84) |
| Malignancy | 0.88 (0.64 – 1.22) | 0.83 (0.59 – 1.17) | 0.79 (0.55 – 1.12) |
| Dementia | 1.17 (0.78 – 1.73) | 1.16 (0.78 – 1.74) | 1.17 (0.77 – 1.78) |
| Depression | 0.77 (0.55 – 1.09) | 0.79 (0.56 – 1.12) | 0.80 (0.56 – 1.14) |
| Previous stroke | **2.04 (1.59 – 2.63)** | **2.05 (1.58 – 2.65)** | **2.13 (1.74 – 2.77)** |
| Previous acute coronary syndrome | 1.23 (0.94 – 1.60) | 1.20 (0.92 – 1.57) | 1.16 (0.87 – 1.54) |
| Previous embolism | 1.39 (0.97 – 1.99) | 1.35 (0.93 – 1.97) | **1.49 (1.03 – 2.18)** |
| Previous haemorrhagic stroke | 0.50 (0.12 – 2.07) | 0.51 (0.12 – 2.13) | 0.29 (0.04 – 2.13) |
| Previous GI bleeding | 1.38 (0.86 – 2.21) | 1.50 (0.94 – 2.40) | 1.39 (0.84 – 2.31) |
| Previous other bleeding | 0.89 (0.69 – 1.62) | 0.91 (0.70 – 1.20) | 0.97 (0.73 – 1.27) |
| ***Events in the first year of follow-up*** |  |  |  |
| Ischemic stroke | **3.33 (1.90 – 5.83)** | **3.40 (1.96 – 5.91)** | **3.14 (1.73 – 5.71)** |
| Hemorrhagic stroke | 2.21 (0.69 – 7.11) | 1.98 (0.60 – 6.47) | 1.84 (0.45 – 7.61) |
| Gastrointestinal bleeding | 1.83 (0.66 – 5.07) | 1.20 (0.38 – 3.73) | 0.64 (0.09 – 4.45 |
| Major gastrointestinal bleeding | 0.00 (0.00 – 2.85) | 0.00 (0.00 – 3.27) | 0.00 (0.00 – 1.16) |
|  |  |  |  |
| ***Model information*** | | | |
| *n* | 28,339 | 28,129 | 26,452 |
| *Deaths* | 369 | 364 | 343 |
| *Person-years, n* | 40,943 | 40,589 | 38,394 |
| *Mean follow-up, years* | 1.44 | 1.44 | 1.45 |
| *Rate, 100 person/years (95%CI)* | 0.90 (0.81 – 1.00) | 0.89 (0.81 – 0.99) | 0.89 (0.80 – 0.99) |
| *Harrell’s C Statistic* | 0.671 | 0.679 | 0.671 |
| PDC: Proportion of days covered; PDC80: Percentage of patients with PDC ≥ 80%. PD: Prescription-dispensation cohort; D1: Dispensation-only cohort with at least 1 refill; D2: Dispensation-only cohort with at least two refills. HR: Hazard Ratio; | | | |

| **Table S4. Association between adherence (PDC≥80%) and hospital admission for bleeding episode according to the different approaches to estimate PDC. Cox proportional hazards regression models** | | | |
| --- | --- | --- | --- |
|  | **PD Cohort**  **HR (95%CI)** | **D1 Cohort**  **HR (95%CI)** | **D2 Cohort**  **HR (95%CI)** |
| ***Adherence*** | | | |
| PDC ≥80% (vs. PDC<80%) | 1.04 (0.68 – 1.58) | 0.96 (0.79 – 1.15) | 0.86 (0.71 – 1.04) |
| ***Sociodemographic characteristics*** | | | |
| Women (vs. Men) | 1.02 (0.86 – 1.21) | 1.04 (0.87 – 1.23) | 1.02 (0.85 – 1.22) |
| Age 65-74y (vs. <65y) | **1.50 (1.04 – 2.15)** | **1.43 (1.00 – 2.05)** | **1.46 (1.10 – 2.10)** |
| Age 75-84y (vs. <65y) | **1.98 (1.38 – 2.84)** | **1.88 (1.31 – 2.68)** | **1.93 (1.34 – 2.78)** |
| Age 85+ (vs. <65y) | **2.27 (1.51 – 3.40)** | **2.16 (1.44 – 3.25)** | **2.20 (1.45 – 3.34)** |
| ***Clinical characteristics and comorbidities at baseline*** | | | |
| Atrial fibrillation (vs. Atrial flutter) | **1.72 (1.07 – 2.76)** | **1.71 (1.07 – 2.74)** | **1.72 (1.06– 2.80)** |
| CHA2DS2-VASC moderate risk (vs. low) | 1.32 (0.47 – 3.72) | 1.37 (0.49 – 3.86) | 1.14 (0.40 – 3.25) |
| CHA2DS2-VASC high risk (vs. low) | 1.75 (0.66 – 4.58) | 1.87 (0.71 – 4.92) | 1.57 (0.60 – 4.15) |
| HAS BLED ≥3 (vs. <3) | 0.94 (0.74 – 1.18) | 0.98 (0.77 – 1.23) | 0.95 (0.75 – 1.21) |
| Heart failure | 1.09 (0.88 – 1.35) | 1.09 (0.88 – 1.35) | 1.10 (0.88 – 1.38) |
| Diabetes | **1.20 (1.02 – 1.42)** | **1.20 (1.02 – 1.42)** | **1.21 (1.02 – 1.44)** |
| Renal disease | 1.24 (0.97 – 1.57) | 1.21 (0.94 – 1.54) | 1.21 (0.94 – 1.56) |
| Hypertension | 1.20 (0.93 – 1.55) | 1.22 (0.95 – 1.57) | 1.19 (0.95 – 1.57) |
| Liver disease | 1.08 (0.77 – 1.50) | 1.08 (0.77 – 1.50) | 1.06 (0.75 – 1.50) |
| Malignancy | 1.15 (0.92 – 1.45) | 1.14 (0.90 – 1.44) | 1.11 (0.87 – 1.41) |
| Dementia | 1.23 (0.90 – 1.69) | 1.22 (0.89 – 1.68) | 1.23 (0.89 – 1.71) |
| Depression | 0.91 (0.71 – 1.18) | 0.91 (0.71 – 1.18) | 0.91 (0.70 – 1.18) |
| Previous stroke | 1.04 (0.82 – 1.31) | 1.04 (0.82 – 1.32) | 1.06 (0.83 – 1.35) |
| Previous acute coronary syndrome | 1.19 (0.96 – 1.46) | 1.17 (0.95 – 1.45) | 1.19 (0.96 – 1.47 |
| Previous embolism | 1.15 (0.86 – 1.55) | 1.18 (0.87 – 1.60) | 1.24 (0.92 – 1.68) |
| Previous haemorrhagic stroke | 1.34 (0.59 – 3.02) | 1.40 (0.62 – 3.15) | 1.61 (0.72 – 3.61) |
| Previous GI bleeding | 1.88 (1.38 – 2.56) | 1.95 (1.43 – 2.67) | 1.95 (1.41 – 2.68) |
| Previous other bleeding | 1.40 (1.15 – 1.69) | 1.40 (1.16 – 1.70) | 1.42 (1.17 – 1.73) |
| ***Events in the first year of follow-up*** |  |  |  |
| Ischemic stroke | 2.07 (1.16 – 3.71) | 2.13 (1.19 – 3.80) | 2.21 (1.24 – 3.96) |
| Haemorrhagic stroke | 0.65 (0.12 – 3.36) | 0.31 (0.04 – 2.67) | 0.65 (0.09 – 4.33) |
| Gastrointestinal bleeding | 4.41 (2.31 – 8.46) | 4.05 (2.05 – 7.98) | 4.27 (2.11 – 8.64) |
| Major gastrointestinal bleeding | 1.41 (0.56 – 3.52) | 1.43 (0.56 – 3.67) | 1.87 (0.72 – 4.82) |
|  |  |  |  |
| ***Model information*** | | | |
| *n* | 28,339 | 28,128 | 26,451 |
| *Deaths* | 593 | 587 | 568 |
| *Person-years, n* | 40,792 | 40,439 | 38,248 |
| *Mean follow-up, years* | 1.44 | 1.44 | 1.45 |
| *Rate, 100 person/years (95%CI)* | 1.45 (1.34 – 1.57) | 1.45 (1.34 – 15.74) | 1.48 (1.37 – 1.61) |
| *Harrell’s C Statistic* | 0.658 | 0.660 | 0.661 |
| PDC: Proportion of days covered; PDC80: Percentage of patients with PDC ≥ 80%. PD: Prescription-dispensation based cohort; D1: Dispensation-only cohort with at least 1 refill; D2: Dispensation-only cohort with at least two refills. HR: Hazard Ratio; | | | |

| **Table S5. Proportion of adherent patients (PDC≥80) according to prescription-dispensation and dispensation- only designs. Only new users recruited after the Apixaban market release.** | | | | | |
| --- | --- | --- | --- | --- | --- |
|  | **Acenocoum.** | **Apixaban** | **Dabigatran** | **Rivaroxaban** | **Total OAC** |
| ***Prescription-dispensation cohort (patients with at least 1 prescription)*** | | | | | |
| n | 16,776 | 2,075 | 1,551 | 2,261 | 22,663 |
| PDC80 (95CI) | 92.39  (91.97-92.78) | 93.59  (92.45-94.57) | 91.30  (89.79-92.60) | 92.22  (91.04-93.25) | 92.40  (92.05 – 92.74) |
| ***Dispensation-only cohort (patients with at least 1 dispensation)*** | | | | | |
| n | 16,672 | 2,047 | 1,529 | 2,225 | 22,473 |
| PDC80 (95CI) | 76.00  (75.35 – 76.64) | 84.86  (83.24-86.35) | 73.45  (71.17– 75.60) | 81.75  (80.09 – 83.30) | 77.20  (76.65– 77.75) |
| ***Dispensation-only cohort (patients with at least 2 dispensations separated by 6 months)*** | | | | | |
| n | 15,704 | 1,923 | 1,340 | 2,039 | 21,006 |
| PDC80 (95CI) | 80.65  (80.03-81.26) | 90.22  (88.81-91.47) | 83.73  (81.65-85.61) | 89.11  (87.68-90.39) | 82.54  (82.03-83.06) |
| PDC: Proportion of Days Covered; CI: Confidence interval; PDC80: % of patients with PDC values equal or above 80%; | | | | | |
